# Essentializing the binary self: individualism and collectivism in cultural neuroscience

**DOI:** 10.3389/fnhum.2013.00289

**Published:** 2013-06-21

**Authors:** M. Martínez Mateo, M. Cabanis, J. Stenmanns, S. Krach

**Affiliations:** ^1^Department of Philosophy, Goethe University FrankfurtFrankfurt am Main, Germany; ^2^Department of Psychiatry, Philipps-University MarburgMarburg, Germany; ^3^Department of Human Geography, Goethe University FrankfurtFrankfurt am Main, Germany

**Keywords:** individualism-collectivism, binarity, postcolonial studies, cultural neuroscience, self-views

## Abstract

Within the emerging field of cultural neuroscience (CN) one branch of research focuses on the neural underpinnings of “individualistic/Western” vs. “collectivistic/Eastern” self-views. These studies uncritically adopt essentialist assumptions from classic cross-cultural research, mainly following the tradition of Markus and Kitayama (1991), into the domain of functional neuroimaging. In this perspective article we analyze recent publications and conference proceedings of the 18th Annual Meeting of the Organization for Human Brain Mapping (2012) and problematize the essentialist and simplistic understanding of “culture” in these studies. Further, we argue against the binary structure of the drawn “cultural” comparisons and their underlying Eurocentrism. Finally we scrutinize whether valuations within the constructed binarities bear the risk of constructing and reproducing a postcolonial, orientalist argumentation pattern.

At the 18th Annual Meeting of the Organization for Human Brain Mapping which was held in Beijing (June 10–14, 2012) the official program was amended by the philosophical supplement “Entering the Mind's I: Some reflections on the Chinese notion of self.” The supplement begins by explaining that the “concept of the individual as outlined by Western philosophy finds its most successful and most immediate conceptual and visual transposition in the work The Vitruvian Man by Leonardo [da Vinci].” The authors of this supplement pursue by stating that “No iconographic representation could be more antithetical to the concept of an individual characterized by the entirety of Chinese philosophy and culture (…)” (Lietti, 2012).

During the conference various other contributions, symposia [e.g., “Imaging the sociocultural human brain” by Gao (2012)], i-poster presentations, or posters addressed “culturally” tuned ways of understanding the self. In these presentations the neural basis of “individualistic/Western” and “collectivistic/Eastern” “cultures” and their way of treating the self were discussed in comparison based on new insights from functional neuroimaging.

But what does it mean to presume a “culturally” imprinted self? And what are the implications of considering two seemingly complementary groups with putatively opposed world- and self-views? The classic review of “cross-cultural” research by Markus and Kitayama (1991) represents the primary inspiration for actual neuroimaging work on “East/West” comparisons. We argue that, by doing so, assumptions implied in classic cross-cultural research are adopted to the functional neuroimaging community without being scrutinized. “Psychological” findings about “cultural differences” are thereby translated onto a “biological” level treating “culture” as a characteristic which can be read out from the body. By means of neuroimaging technology the simplifications of “culture” inherent to many cross-cultural psychological studies receive additional support as cultural differences can now be fostered by biological “evidence.”

Here we elaborate why such neuroscientific findings bear the risk of constructing and reproducing essentialist (1), binarized (2), and Eurocentric (3) ways of thinking and acting which follow a postcolonial and orientalist tradition (4). These four dimensions build the frame for the current analysis. They all refer to specific traditions of critique which originate from philosophy and social science and which will be introduced in more detail in the respective sections of this manuscript.

The endeavor to studying “cultural” phenomena by using functional MRI started only in the last decade (Chiao, 2009; Han and Northoff, 2009; Vogeley and Roepstorff, 2009; Kitayama and Park, 2010; Losin et al., 2010; Bao and Pöppel, 2012; Han et al., 2013; Rule et al., 2013). Since the year 2000 the number of publications in the cultural neurosciences (CN) has increased tremendously. Although particular concepts of “culture” are implied, these are only rarely explicitly addressed (Martínez Mateo et al., 2012, 2013). Within the field of CN, however, a particular branch has focused on “culturally” tuned ways of understanding the self [see Martínez Mateo et al. (2012) for a review on different branches in CN]. For the purpose of the present article we searched (i) peer-reviewed English language manuscripts of original functional MRI studies indexed in large databases (e.g., Google Scholar; PubMed) and (ii) abstracts published in the this year's OHBM abstract book [http://www.humanbrainmapping.org/files/2012MeetingFiles/OHBM_2012-Abstractsfinal.pdf] which addressed the neural correlates of the self or self-concepts such as individualism and collectivism in a “cultural” context using cerebral blood flow imaging techniques such as fMRI or fNIRS. Overall, 10 manuscripts and 10 conference abstracts fulfilled these criteria and thus, formed the data pool for the present analysis.

From these publications we extracted the aforementioned four fundamental dimensions which we problematize by briefly discussing their immanent assumptions, their implications and consequences.

## Essentialism

Essentialism refers to the idea that the concept of e.g., the “Chinese” or the “Westerner” has its own real and discrete essence (Narayan, 1998). This view does not consider the historical and socio-political contexts in which such concepts are used. Many of the analyzed studies take such an essentialist understanding of “culture” as their starting point for the elaboration of their research hypotheses as well as for the justification of observed neural differences between “cultural” groups. Wang and colleagues e.g., speak of “collectivistic brains” (Wang et al., 2012) when referring to their Chinese sample, while Sul and colleagues label their Chinese participants “collectivistic people” having an “interdependent self, typically found in collectivistic cultures (e.g., East Asia)” (Sul et al., 2012). Other variations on this theme, which likewise view an individual as a representative for a “cultural” totality, assign their study participants a “Chinese self” (Zhang et al., 2006) or a group membership in “Eastern “Face” cultures such as Chinese” (Honghong et al., 2012). In these examples, “cultural identity” is made essential. Said criticizes such essentialism as it implies violence toward the presumed “members” of such categories as they are forced to correspond to this conceptual essence: “An Oriental man was first an Oriental and only second a man” (Said, 1979). Balibar (2011) and Taguieff (1991) claim that even without touching the concept of “race,” an essentialist idea of one “cultural identity” follows a culturalistic, “neo-racist” argumentation pattern. The term “neo-racism” points out that approaches to “cultural diversity,” even though having overcome a system of “racial” hierarchies, share the idea of clearly definable “cultural” demarcations. Thus, as soon as persons are reduced to their “cultural belongings,” and this forms the basis for interpreting their behavior, there is no significant difference between the concept of “race” and the concept of “culture.”

## Binarity

“Cross-cultural” research necessarily involves comparisons between “cultural” groups. Interestingly, within the analyzed CN studies all comparisons follow a solely binary structure assuming “cultural” values as being opposed to each other. The starting point of all analyzed studies is the distinction between “Europe” and “Asia,” “Occident” and “Orient” or “East” and “West.” Based on this dichotomous world-view, “cultural” values are linked to either one of these binary poles. For example, “independent cultures (such as the German)” (De Greck et al., 2012) are paralleled to “East Asian cultures [which] generally show more inter-dependent self-views” (Korn et al., 2012), “Western societies [which] are individualistic, promoting the value of independence, individual goals and rights” are opposed to “Eastern societies [which] are collectivistic, promoting the value of interdependence, group goals and duties.” (Vizioli et al., 2012) or finally, group-agency is distinguished from self-agency as in the following quotation from Kobayashi et al. (2007): “Increasing evidence from socio-psychological studies suggests that Japanese and other Asian cultures encourage the use of group-agency more than individualistic self-agency […]. The diminished activity in the TPJ area in Japanese adults and children during the ToM tasks might represent the demoted sense of self-other distinction in the Japanese culture.”

Some of the analyzed studies circumvent the direct comparison of “biologically” or “geographically” predefined groups and use “cultural priming” methods instead. Here, “symbols (e.g., the American flag vs. a Chinese dragon), legendary figures from folklore or popular cartoons (e.g., Superman vs. Stone Monkey), famous people (e.g., Marilyn Monroe vs. a Chinese opera singer), and landmarks (e.g., the Capitol Building vs. the Great Wall)” (Hong et al., 2000) are used in order to prime “cultural frameworks” (Sui et al., 2013) in “bicultural” individuals. By this, the authors aim to activate a “corresponding cultural meaning system” (Hong et al., 2000) and trigger “prototypically interdependent” or “independent” behaviors, respectively (Markus and Kitayama, 2010). However, such “cultural priming” obviously depends on simplistic presumptions about “biculturality,” viewing both “cultures” as similarly dozing in one's identity waiting to be woken up. The essence of this awakening is found in stereotypical attributions about the respective “cultural” contexts (e.g., China being represented by the Great Wall or “the West” being represented by the Statue of Liberty). Moreover, the binary structure is still at the core of such priming studies. Therefore, all results have to confirm the underlying binary self-construal (e.g., “independent” or “interdependent”). Furthermore, “cultural” representation is valued regarding its fit to the expected, “mandated way” [“Within a given culture, however, individuals will vary in the extent to which they are good cultural representatives and construe the self in the mandated way” (Markus and Kitayama, 1991)]. The results, ultimately, cannot but reproduce the binary structure and thereby strengthen the simplistic understanding of “cultural frameworks.” All internal heterogeneities within a construed population are thereby resolved in favor of this one central distinction. Further, we view this to be problematic as binary structures always imply hierarchies between the two poles^1^.

## Eurocentrism

Neatly tied to the latter reasoning, i.e., that binarity implies and reproduces structures of dominance, is that a Eurocentric perspective is promoted in the analyzed studies. As the historian Dipesh Chakrabarty argues in his book *Provincializing Europe*, up until the present, Eurocentric structures remain mostly unchallenged. Hence, the figure of Europe remains a cultural bench-mark for difference and deviance and functions as a “silent referent” (Chakrabarty, 2000). Thereby the “Western perspective” is taken as the “gold standard” (Oyserman et al., 2002). Accordingly, in many incidents authors contrast “non-Western” values or identities with their “Western” counterparts: e.g., “The essential aspect of this view involves a conception of the self as an autonomous, independent person; we thus refer to it as the independent construal of the self. Other similar labels include individualist, egocentric, separate, autonomous, idiocentric, and self-contained. We assume that, on average, relatively more individuals in Western cultures will hold this view than will individuals in non-Western cultures.” (Markus and Kitayama, 1991) or “Many non-Western cultures do not construe behaviors as personal and intentional” (Kobayashi and Temple, 2009). “Western” views on the self are further linked to modernity and freedom [e.g., “In more individualistic cultures, the expression of thoughts, preferences, and needs is viewed as an expression of selfhood, and thus, freedom of expression is a sign of individual freedom and an independent self.” (Sherman et al., 2009)], which underscores an implicit valuation of this part of the binary structure.

## Postcolonial and orientalist view on the self

We argue that the above mentioned valuations within the constructed binarities fit in a postcolonial, orientalist argumentation-pattern. The term “post-colonial” usually refers to a historical period of formal independence *after* colonialism. However, within the humanities and social sciences, the term postcolonial (spelled without a hyphen) refers both to a historical process with innumerable repercussions continuing to the present day and to its critical analysis. Against this background, postcolonial studies critically engage with and challenge the seemingly fixed and clearly persistent, yet changing, colonial binarities such as modern/pre-modern or civilized/uncivilized and their contemporary reproductions [see e.g., Said, 1979]. From a postcolonial perspective, the centuries of European expansion are understood as a unique, global process, which has been socially, culturally, and materially inscribed into the present structure of the world. The politics of binarity, deeply ingrained in colonialism, allowed a privileged order, whereby Europe represented itself as the enlightened and modern self. Modernity and Enlightenment imagined as products of European autogenesis, hence legitimated the European civilizing mission.

Orientalism, as defined by Said (1979), refers to the production of knowledge about the “East” or the “Orient” as complementary to the “West” or the “Occident”. Research in this context, i.e., orientalism, is based on stereotypes and contains racist implications, dating back to the British colonial politics from the beginning of the last century (Said, 1979). The following quotation as stated in a direct context of colonial regime would provide such an example for orientalist reasoning: “The European is a close reasoner; his statements of fact are devoid of any ambiguity; he is a natural logician, albeit he may not have studied logic; (…) The mind of the Oriental, on the other hand, like his picturesque streets, is eminently wanting in symmetry” [Lord Cromer, 1908, quoted from Said (1979)]. This fits into the idea of an Eastern holistic (respectively undistinguished, synthetical, and Oriental) self, contrasted to a Western analytical (respectively independent, rational, and European) self, which is put forward by Markus and Kitayama: “[…] a holistic view is in opposition to the Cartesian, dualistic tradition that characterizes Western thinking and in which the self is separated from the object and from the natural world.”

Based on this continuity, the quoted CN studies must be regarded as part of a postcolonial, orientalist history and their results cannot be considered as neutral truths, but rather as perspectives which are deeply rooted in colonial knowledge production. These perspectives reinforce Western dominance in a postcolonial situation and miss the opportunity to illustrate other possible views on the world. This is a structure which remains in force, independently of the individual valuations of this binarity by the researchers. The bifid order itself and not necessarily the valuation of one of the two poles are therefore problematized. Notwithstanding of the contextual setting or “cultural” background of a neuroscientist, we plead for taking into account the colonial legacies of these epistemological orderings of the world and their manifold contemporary repercussions.

## Conclusion

In this *Perspective* we analyzed both current poster abstracts from the 18th Annual Meeting of the OHBM in Beijing and scientific research published in peer-reviewed journals addressing the neural foundations of “culturally” shaped ways of treating the self. Our aim was to disclose their presumed and often unexamined implications. We elaborated that the comparisons within cultural neuroscience studies referring to the self are not based on postulated “naturally given facts,” but result from a (post-)colonial argumentation pattern. With Balibar (2011) and Taguieff (1991) we argue that drawn “cultural” comparisons underlie the risk of reproducing Eurocentric structures of domination, implying neo-racist, essentialized categories and stereotypes. Functional neuroimaging techniques thereby offer an easy and tempting option to translate former “psychological” findings on “cultural differences,” as based on the findings in the review by Markus and Kitayama, onto a “biological” level. The danger of a Looping Effect (Hacking, 1995) becomes evident, since the produced knowledge, particularly knowledge based on “hard data” from the neurosciences, has the status of great scientific truth in society.

Although often advocated in our, in part controversial, poster discussions at the Annual Meeting, we claim that the obtained functional MRI data is never neutral (Martínez Mateo et al., 2013). Both at the level of hypotheses generation and at the level of data interpretation, all research is influenced by specific socio-political and historical contexts. In this respect we argue that all quoted CN studies referring to the self are rooted in a specific context which defines the relevant research questions and topics and the way of interpretation. This context is traversed by social circumstances, political interests, and imbalances of power (Martínez Mateo et al., 2012).

Another critique of ours relates to the fact that scientific discourses always run the risk of self-referential biasing, which is especially fostered by the fact that almost all CN research ideas are based on one single influential review on cross-cultural psychological findings (Markus and Kitayama, 1991). This self-referentiality of CN studies generates and, at the same time, confirms scientific facts making it difficult to question the underlying premises.

In sum, during our poster discussions at the Annual Meeting of the OHBM it became evident that this debate is overdue to entry the neuroscience community. We believe that critical analyses regarding (i) the way research questions are formed, as well as, (ii) how research findings are projected back into society, are as yet neglected necessities.

### Conflict of interest statement

The authors declare that the research was conducted in the absence of any commercial or financial relationships that could be construed as a potential conflict of interest.

## Footnotes

^1^The idea that binarities always have an inherent hierarchical structure follows a critique articulated by Jacques Derrida among others: “We are not dealing with the peaceful coexistence of a vis-à-vis, but rather with a violent hierarchy. One of the two terms governs the other (axiologically, logically, etc.), or has the upper hand” (Derrida, 1981).
